# It’s all about control: Sense of control mediates the relationship between physical activity and mental health during the COVID-19 pandemic in Germany

**DOI:** 10.1007/s12144-021-02303-4

**Published:** 2021-10-20

**Authors:** Lena-Marie Precht, Jürgen Margraf, Jan Stirnberg, Julia Brailovskaia

**Affiliations:** Mental Health Research and Treatment Center, Department of Clinical Psychology and Psychotherapy, Ruhr-Universität Bochum, Massenbergstr. 9-13, 44787 Bochum, Germany

**Keywords:** COVID-19, Physical activity, Sense of control, Positive mental health, Depression, anxiety, stress

## Abstract

COVID-19-related burden has a significant impact on mental health and has led to an increase of depression, anxiety, and stress symptoms. Physical activity has been suggested to mitigate the negative effects of the pandemic and to foster mental health. The present study aimed to investigate, whether sense of control might mediate the supposed beneficial effects of physical activity on positive (PMH) and negative mental health (NMH) in unpredictable extraordinary situations. Data were assessed in a sample of 568 students (*M*_age_ = 19.90, *SD*_age_ = 4.52) from Germany via an online survey in fall 2020. Mediation analyses revealed that sense of control mediated the relation between physical activity and PMH as well as depression, anxiety, and stress symptoms, respectively. The findings indicate that physical activity may be a promising strategy for fostering sense of control and thus mental health. Due to its practical implications and practicability, engagement in physical activity could be an effective way to reduce the NMH consequences of the current COVID-19 situation, and therefore should be addressed in actions for long-term prevention and intervention.

## Introduction

On March 11, 2020, the World Health Organization (WHO) declared the outbreak of the coronavirus disease (COVID-19; caused by the severe acute respiratory syndrome coronavirus 2, SARS-CoV-2) as a pandemic (World Health Organization, 2020a). The following governmental induced restrictive measures for the prevention of its rapid spread – including physical distancing measures, movement restrictions, and wearing of face masks (Tso & Cowling, 2020; World Health Organization, 2020b) – have significantly changed people’s everyday life. While some individuals adapted to the situation and tried to make the best of it, others experienced it as a heavy burden (Brailovskaia & Margraf, 2020). As a consequence, mental health declined (Fruehwirth et al., 2021; Pierce et al., 2020). A recent meta-analysis, based on samples from all over the word, revealed a prevalence of 33.7%, 31.9%, and 29.6% for depression, anxiety, and stress, respectively (Salari et al., 2020).

Anxiety resulting from the pandemic outbreak (Liu et al., 2020), loneliness due to social distancing and isolation (Galea et al., 2020), uncertainties regarding the future (Paredes et al., 2021), and feelings of lack of control (Šrol et al., 2021) have a significant impact on mental health. Traditionally, mental health has been defined as the mere absence of mental illness. However, in recent years it has been recognized that the absence of mental disorders is not the same as the presence of mental health (Keyes, 2005; Lukat et al., 2016). Therefore, mental health is often considered as two interrelated but separate unipolar dimensions, which can be present simultaneously (Keyes, 2005): positive mental health (PMH) and negative mental health (NMH). While PMH comprises facets of subjective and psychological aspects of well-being, NMH refers to mental health problems, psychopathology, or negative well-being (Lukat et al., 2016). In order to reduce the risk of developing mental health problems and to support mental and psychosocial well-being, general advice was provided to the population, e.g., staying in contact with family and friends, limiting the amount of COVID-19 information sources, and maintaining daily routine (Fiorillo & Gorwood, 2020; World Health Organization, 2020c). In addition, maintaining regular physical activity such as jogging, cycling, or swimming has been proposed as an important strategy for mitigating the negative effects of the pandemic on physical and mental health (Brailovskaia et al., 2021; Chen et al., 2020).

Considering physical health, recent research suggests that physical activity can enhance immune defense and reduce the harmful effects of stress on immunity, may be protective for lung function, has the potential to reduce the severity of COVID-19 infections, and improves the immune response to vaccination (Sallis & Pratt, 2020; Simpson & Katsanis, 2020; Woods et al., 2020). Regarding mental health, physical activity is generally known for enhancing self-esteem and resilience to stressful experiences as well as reducing depression and anxiety (Eime et al., 2013; Rebar et al., 2015). In terms of the pandemic, recent studies demonstrated that physical activity could foster mood and well-being (Brand et al., 2020; Maugeri et al., 2020) and is negatively related to perceived burden caused by COVID-19 (Brailovskaia et al., 2021). In addition, some evidence indicates, that physical activity might boost self-efficacy, feelings of mastery, and sense of control which in turn could contribute to an increase of PMH and a decrease of NMH (Bailey et al., 2013; Mikkelsen et al., 2017).

Sense of control is an important personal (coping) resource, that describes the belief that the events and conditions in one’s life are controllable by own actions rather than being a consequence of luck, chance, or fortune (Glavin & Schieman, 2014; Pearlin & Schooler, 1978). Persons with a high sense of control effectively cope with stressors by trying alternative actions if their current behavior does not contribute to a functional solution of the stressful situation (Ross & Mirowsky, 2013). Accordingly, this resource is associated with less distress and a lower risk for mental disorders, e.g., substance use disorder (Kiecolt et al., 2009). Various studies demonstrated relations between sense of control and PMH outcomes (for review see e.g., Ross & Mirowsky, 2013). Especially in extraordinary situations, which are characterized by lots of uncertainties (Šrol et al., 2021), sense of control can be of significant importance. Presumably, some people rely on maladaptive strategies to compensate the reduced sense of control, e.g., by adopting conspiracy beliefs (Šrol et al., 2021), engaging in intensive social media usage (Brailovskaia & Margraf, 2021), and problematic internet usage in general (Islam et al., 2020; Lemenager et al., 2020). Moreover, a recent study found a positive effect of sense of control on emotional well-being (Yang & Ma, 2020), suggesting that (regaining) sense of control might reduce stress, anxiety, and depressed mood.

Considering the presented background, it is important to identify and foster factors that boost sense of control and thereby can alleviate the detrimental effects of the pandemic outbreak on mental health (Yang & Ma, 2020). While it was already shown that PMH (Brailovskaia & Margraf, 2020), higher trust in institution’s response to the pandemic (Šrol et al., 2021) and perceived knowledge (Yang & Ma, 2020) are positively related to sense of control, the main aim of the present study was to investigate the relationship between physical activity and sense of control, which is supposed to be associated with mental health. For a complete mental health assessment according to the dual-factor model (e.g., Keyes, 2005), PMH (Lukat et al., 2016) as well as NMH, operationalized as depression, anxiety, and stress symptoms (Lovibond & Lovibond, 1995), were considered altogether.

Based on this framework, following hypotheses are presumed. As research considering physical activity during COVID-19 described positive relations with mental health (e.g., Faulkner et al., 2021), physical activity was assumed to be positively related to PMH (Hypothesis 1a) and negatively related to symptoms of depression (Hypothesis 1b), anxiety (Hypothesis 1c), and stress (Hypothesis 1d). Furthermore, there is the assumption, that physical activity might foster the individual sense of control, e.g., by the improvement of one’s performance or the achievement of self-imposed physical goals (Bailey et al., 2013). Therefore, it was expected that there is a positive link between physical activity and sense of control (Hypothesis 2). Moreover, sense of control was presumed to be positively related to PMH (Hypothesis 3a) and negatively related to symptoms of depression (Hypothesis 3b), anxiety (Hypothesis 3c), and stress (Hypothesis 3d). Finally, the relations between physical activity and PMH as well as symptoms of depression, anxiety, and stress were hypothesized to be mediated by sense of control (Hypotheses 4a – 4d).

## Methods

### Procedure and Participants

The sample consisted of 568 participants (69% women; *M*_*age*_ (*SD*_*age*_) = 19.90 (4.52), range: 16-66) who were currently enrolled at a large university in the Ruhr region in Germany. Data were collected between October and November 2020, when measures for preventing the spread of COVID-19 – including physical distancing measures, limitation of people gathering, shutdown of specific institutions, and wearing of face masks – were still present (Bundesministerium für Gesundheit, 2021). The online survey took place within the frame of the ongoing Bochum Optimism and Mental Health (BOOM) program, that investigates risk and protective factors of mental health (Maercker et al., 2015; Schönfeld et al., 2017). All participants have been invited to participate by e-mail. They were properly instructed and gave informed consent via an online form. Participation was voluntary and compensated by course credits. The study has received ethical approval from the responsible Ethics Committee. There were no missing data. Power analyses using the G*Power program, version 3.1 (Faul et al., 2009) indicated that the sample size was sufficient for valid results (power > .80, *α* = .05, effect size: *f*^*2*^ = .02; cf., Cohen, 1992).

### Materials

#### Physical activity

The frequency of physical activity was assessed by the item “How frequently did you engage in physical activity (e.g., jogging, cycling) in the last 12 months?”. It was rated on a 5-point Likert-type scale (1 = *never*, 5 = *four times a week or more*) and was shown to be a reliable and valid instrument to measure physical activity (Brailovskaia et al., 2021; Milton et al., 2011).

#### Sense of control

According to Brailovskaia and Margraf (2021) sense of control was measured with the two items “Do you experience important areas of your life (i.e., work, free-time, family, etc.) to be uncontrollable, meaning that you cannot or barely can influence them?” and “Do you experience these important areas of your life as unpredictable or inscrutable?”. They were rated on a 5-point Likert-type scale ranging from 0 (*not at all*) to 4 (*very strong*), so that higher sum scores indicate lower sense of control. Current scale reliability was *α* = .76.

#### Positive mental health

The unidimensional Positive Mental Health Scale (PMH-scale; Lukat et al., 2016) served for the assessment of PMH. The nine items (e.g., “I enjoy my life”) were rated on a 4-point Likert-type scale (0 = *do not agree*, 3 = *agree*), with higher sum scores indicating higher levels of PMH. Current scale reliability was *α* = .91.

#### Negative mental health

NMH was measured by the Depression Anxiety Stress Scales 21 (DASS-21; Lovibond & Lovibond, 1995). The questionnaire contains seven items for each subscale: depression (e.g., “I couldn’t seem to experience any positive feeling at all”), anxiety (e.g., “I felt scared without any good reason”), and stress symptoms (e.g., “I tended to over-react to situations”). The items were rated on a 4-point Likert-type scale (0 = *did not apply to me at all*, 3 = *applies to me very much or most of the time*). Higher sum scores indicate higher symptoms of depression, anxiety, and stress, respectively. Current scale reliabilities were *α* = .92 for depression, *α* = .83 for anxiety, and *α* = .87 for stress.

### Statistical Analyses

Statistical analyses were conducted using SPSS 27 and the macro PROCESS version 3.5.3 (Hayes, 2020). After descriptive statistics, the relationship between physical activity, sense of control, PMH, and the NMH constructs (i.e., symptoms of depression, anxiety, and stress) was assessed by zero-order bivariate correlations. Next, four mediation models (PROCESS: model 4) were calculated. Each model included physical activity as predictor and sense of control as mediator. PMH (model 1), depression (model 2), anxiety (model 3), and stress (model 4), respectively, served as outcome. Age and gender, which were considered as control variables, were included as covariates. In all mediation models, the basic relationship between predictor and outcome was referred to as *c* (total effect), the path of predictor to mediator as *a* and the path of mediator to outcome was referred to as *b*. The combined effect of paths *a* and *b* represented the indirect effect (*ab*), and the direct effect of predictor to outcome after the inclusion of the mediator in the model was referred to as *c’*. The mediation effect was assessed by bootstrapping procedure (10,000 samples), which provides percentile bootstrap confidence intervals (95% CI).

## Results

Table 1 displays the descriptive statistics of the investigated variables and their correlations. The means of the NMH constructs depression, anxiety, and stress were 7.24 (disorder cut-off ≥ 10), 5.58 (disorder cut-off ≥ 6), and 8.62 (disorder cut-off ≥ 10), respectively. All investigated constructs were significantly correlated (*p* < .001) (see Table 1). Physical activity was positively related to PMH and negatively related to sense of control (whereupon higher values indicate a lower sense of control) as well as to symptoms of depression, anxiety, and stress. Sense of control was negatively related to PMH and positively related to symptoms of depression, anxiety, and stress. PMH was negatively related to all NMH constructs. The NMH constructs were positively correlated.

**Table 1:** Descriptive statistics and correlations of physical activity, sense of control, positive mental health (PMH), depression, anxiety, and stress symptoms.

	*M (SD)*	*Min–Max*	(2)	(3)	(4)	(5)	(6)
(1) Physical activity	3.32 (1.22)	1–5	-.177**	.276**	-.237**	-.193**	-.145**
(2) Sense of control	3.26 (1.95)	0–8		-.524**	.522**	.455**	.482**
(3) PMH	16.60 (5.82)	1–27			-.762**	-.543**	-.621**
(4) Depression	7.24 (5.82)	0–21				.610**	.649**
(5) Anxiety	5.58 (4.80)	0–20					.748**
(6) Stress	8.62 (5.11)	0–21					

*Note*. *N* = 568; *M* = mean, *SD* = standard deviation, *Min* = minimum, *Max* = maximum; Sense of control: the higher the value, the lower sense of control; **p < .001.

**Fig. 1:**
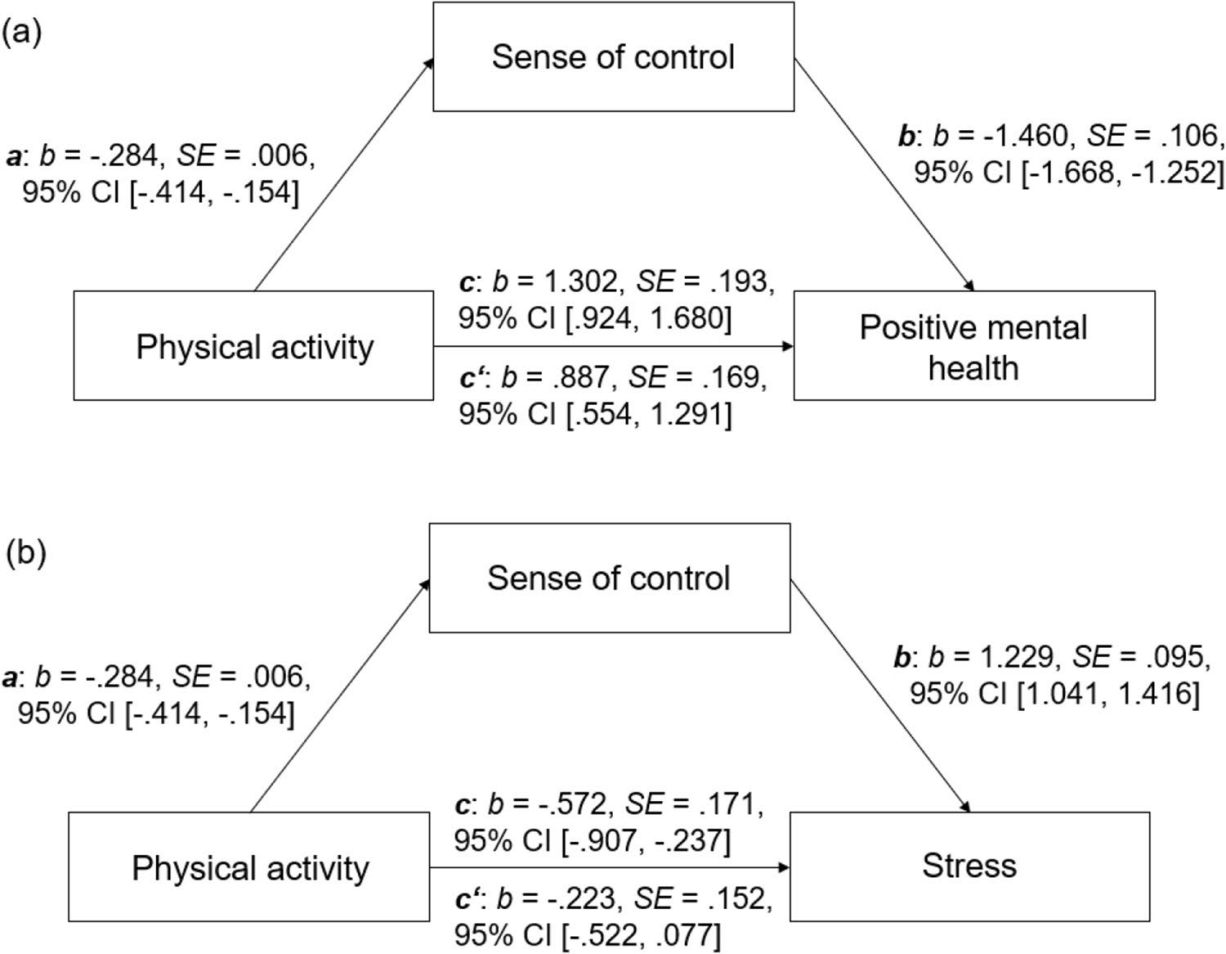
(a) Mediation model including physical activity (predictor), sense of control (mediator) and positive mental health (outcome); (b) Mediation model including physical activity (predictor), sense of control (mediator) and stress (outcome). *Note. c* = total effect, *c’* = direct effect; *b*: standardized regression coefficient, *SE*: standard error, CI: confidence interval.

Figure 1 shows the results of the bootstrapped mediation analyses with physical activity as predictor, sense of control as mediator and (a) PMH as well as (b) NMH, namely stress symptoms, as outcomes. The results of all four mediation models that are presented in Table 2 indicate that sense of control mediates the relationship between physical activity and PMH as well as depression, anxiety, and stress symptoms, respectively. The basic relationships between physical activity and the four outcomes were significant (see Table 2, total effect, *c*: *p* < .001). The link between physical activity and sense of control (path *a: p* < .001) and the associations between physical activity and all four outcomes (path *b*: *p* < .001) were also significant. While the relationship between physical activity and stress was no longer significant after the inclusion of sense of control in the model (direct effect *c’*: *p* = .144), the relationships between physical activity and PMH, depression, and anxiety, respectively, remained significant (*c’*: *p* < .01). However, the total effects were higher than the direct effects (see Table 2). All indirect effects (*ab*) were significant (see Table 2).

**Table 2:** Estimated coefficients of the mediation models with physical activity as predictor, sense of control as mediator as well as positive mental health (PMH), depression, anxiety, and stress as outcomes (controlling for age and gender).

Outcome	Total effect			Direct effect			Indirect effect		
	*c*	*SE*	95% CI	*c’*	*SE*	95% CI	*ab*	*SE*	95% CI
PMH	1.302	.193	[.924, 1.680]	.887	.169	[.554, 1.219]	.415	.108	[.207, .628]
Depression	-1.139	.194	[-1.521, -.758]	-.722	.171	[-1.057, -.386]	-.418	.108	[-.632, -.210]
Anxiety	-.739	.159	[-1.050, -.427]	-.438	.145	[-.722, -.154]	-.301	.078	[-.457, -.154]
Stress	-.572	.171	[-.907, -.237]	-.223	.152	[-.522, .077]	-.349	.090	[-.527, -.173]

*Note*. *N* = 568; *SE* = standard error; CI = confidence interval; all CIs generated with bootstrapping: N = 10,000; *c* = total effect, *c’* = direct effect, *ab* = indirect effect.

## Discussion

Since the beginning of 2020, COVID-19 has spread all over the globe. Although global case and death incidences are recently decreasing (World Health Organization, 2021b) and the access to effective vaccines (World Health Organization, 2021a) seems promising to combat the virus, the end of the pandemic is not yet foreseeable. It still affects daily life and has a large mental health impact not only on vulnerable groups, including healthcare professionals, people with pre-existing mental health issues and those infected with COVID-19, but also on the general population (Tsamakis et al., 2021). In addition, experts forecast that this impact can persist and be long lasting for several years during the post-pandemic time and expect increases in the prevalence of mental disorders and suicide (Kathirvel, 2020). Therefore, it is important to not only strengthen and expand mental health services, namely in terms of full- and semi-inpatient as well as outpatient care, but also to identify and communicate low-threshold prevention and intervention strategies – e.g., the reduction of social media usage or reaching adequate amounts of physical activity (see e.g., Brailovskaia et al., 2020) – that may foster mental health in the general population.

Regular physical activity has been suggested as an important approach for mitigating the negative effects of the pandemic on physical and mental health (Chen et al., 2020; Sallis & Pratt, 2020). Since evidence suggests that physical activity is positively linked to mental health in general (e.g., Mikkelsen et al., 2017) and especially during the pandemic (e.g., Brailovskaia et al., 2021; Brand et al., 2020), and there is also evidence for the importance of sense of control (e.g., Yang & Ma, 2020), the present study investigated the relationship between physical activity and PMH as well as NMH, while considering the role of sense of control. The current results revealed that sense of control mediates the relation between physical activity and PMH as well as depression, anxiety, and stress symptoms, respectively.

As expected, physical activity was positively related to PMH and negatively related to depression, anxiety, and stress (small effect sizes; confirmation of Hypotheses 1a – 1d). This is in line with numerous previously published studies (for review see e.g., Eime et al., 2013; Rebar et al., 2015). But also recently conducted studies reported relations with PMH and NMH with reference to the COVID-19 situation. Across different countries, physical activity was positively related to different forms of well-being and quality of life (Faulkner et al., 2021; Lesser & Nienhuis, 2020; Ozdemir et al., 2020) as well as negatively linked to depression, anxiety, and stress (Jacob et al., 2020; Li et al., 2021; Xiang et al., 2020). Furthermore, it has been shown that physical activity can contribute to less psychological burden and a more adaptive response to the current COVID-19 situation (Brailovskaia et al., 2021).

There are several possible explanations for the beneficial effects of physical activity (Mikkelsen et al., 2017). However, the present study investigated whether physical activity might foster sense of control – a construct directly linked to stress responses and well-being (Steptoe & Poole, 2016) – and thus mental health. As expected, sense of control (higher values indicate lower sense of control) was negatively related to physical activity (small effect; confirmation of Hypothesis 2) and PMH (large effect; confirmation of Hypothesis 3a). Moreover, it was positively related to symptoms of depression (large effect; confirmation of Hypothesis 3b), anxiety, and stress (medium effect sizes; confirmation of Hypotheses 3c and 3d). The confirmation of Hypothesis 2 supports previous studies suggesting that physical activity might foster sense of control (Bailey et al., 2013; Brailovskaia & Margraf, 2021). This could be explained by a possible increase of social networks and community cohesion in team sport or – in times of physical distancing more likely – by experiencing the steady improvement of one’s sportive performance or the achievement of self-imposed physical goals.

The present findings regarding the relations between sense of control and PMH as well as depression, anxiety, and stress complement previous results that emphasized a positive effect of sense of control on emotional well-being (Yang & Ma, 2020) and levels of distress (Ross & Mirowsky, 2013). In addition, individuals with low sense of control are at risk for NMH outcomes like depression, anxiety, and burnout (Maier & Seligman, 2016; Southwick & Southwick, 2018) and tend to engage in maladaptive coping strategies such as overeating, problematic drinking, smoking, or use of psychoactive substances which in turn might lead to acute stress (Ye et al., 2020).

Results of the mediation analyses indicated that sense of control mediates the relation between physical activity and PMH, as well as symptoms of depression, anxiety, and stress (confirmation of Hypotheses 4a – 4d). Hence, it can be assumed, that physical activity might foster or even help to regain sense of control, which can enhance PMH and reduce NMH. This is of specific important in the current COVID-19 situation that is often experienced as a heavy psychological burden (Brailovskaia & Margraf, 2020). The finding, that the direct effects between physical activity and PMH, and symptoms of depression and anxiety, respectively, remained significant after adding sense of control to the model points out to the numerous other mechanisms that can influence the relationship between physical activity and mental health (for review see e.g., Mikkelsen et al., 2017). The no longer significant direct effect in the relation between physical activity and stress symptoms might be explained as following. It seems that stress symptoms could function as another mediator in the relation between physical activity and depression as well as anxiety. In accordance with this, a recent study revealed that stress symptoms may lead to symptoms of depression or anxiety which could later lead to depression (Rodríguez-Hidalgo et al., 2020). This means, that individuals engaging in physical activity could profit from an enhanced sense of control that helps to reduce the experience of stress and thereby, in the long term, the risk for anxiety and depression.

The potential of physical activity to foster sense of control and thereby mental health is of practical importance in two respects. On the one hand, the pandemic outbreak and its consequences have led to an increase of symptoms of depression, anxiety, and stress (Fruehwirth et al., 2021; Pierce et al., 2020). Since aftermath are expected to be long lasting (Kathirvel, 2020), physical activity can be seen as a cost-effective, low-threshold prevention or intervention strategy. As the current study only considered the frequency of physical activity, the present results do not allow inferences concerning advice for the type and further characteristics of physical activity. However, the WHO recommends at least 150 minutes of moderate- or 75 minutes of vigorous-intensity physical activity as well as two sessions of strength training per week (World Health Organization, 2010). Furthermore, recent studies reported running, walking, and cycling (Lesser & Nienhuis, 2020), as well as stretching and resistance training (Xiang et al., 2020) to be beneficial for mental health in times of the pandemic, which is meaningful, since these activities require no specific equipment, only little space and can be practiced at any time at home or outdoors (Chen et al., 2020). Moreover, they can be carried out alone, which seems promising, since team sports that were previously shown to be beneficial for mental health (Chekroud et al., 2018; Johnston et al., 2020) have been banned as well as any other community activities in many countries since the COVID-19 outbreak. On the other hand, sense of control has been proven to be positively related to mental health during the pandemic (Brailovskaia & Margraf, 2020; Yang & Ma, 2020). Furthermore, it can also lead to a reduction of maladaptive coping strategies, like intensive social media usage, as a response to negative feelings (Brailovskaia & Margraf, 2021). Consequently, experiencing a high sense of control is not only beneficial in extraordinary situations such as the outbreak of COVID-19, but also when being confronted with stressors of daily life, e.g., problems with family or friends, or at the workplace. Especially for students who are often exposed to a high pressure to perform, a high level of sense of control can be an important resource for functional coping with the stressful experiences. Thus, the investigation and communication of strategies fostering sense of control are of special importance.

Despite the novelty and the practical importance of the present results, some limitations must be considered. First, the cross-sectional online survey design allows only hypothetical conclusions of causality. Due to social distancing measures in Germany by the time of data collection, experimental investigations were not possible. However, a replication of the present findings in longitudinal experimental studies that address physical activity is eligible. Second, it must be noted that present results are a snapshot of the COVID-19 situation in Germany in fall 2020. Due to the dynamic circumstances of the pandemic as well as different courses and measures for the prevention of the spread of the virus in other countries, it should be investigated whether the relations are replicable in other samples to different time-points. Third, the convenient sample of young university students that are mostly female limits the generalizability of the results. Although age and gender were controlled for in the analyses, future studies should replicate the findings in more age and gender balanced samples. Further, in favor of more representative samples, the consideration of non-WEIRD (western, educated, industrialized, rich, and democratic; Henrich et al., 2010) populations seems worthwhile. Fifth, it is also necessary to investigate at-risk groups like people with pre-existing mental health issues or those who recovered from a COVID-19 infection to find out whether physical activity proves to be an effective intervention strategy under these pre-conditions. Lastly, there were only small effect sizes of the relations between physical activity and sense of control as well as PMH and NMH. Future studies could use well-established, reliable instruments like the International Physical Activity Questionnaire (IPAQ; Craig et al., 2003) for a differentiated assessment of physical activity and investigate further potential moderators (e.g., duration, intensity, or type) of the relationship between the constructs.

In conclusion, the present study provides the first findings from Germany that physical activity might be a promising strategy for fostering sense of control and thereby mental health. This underlines the importance of getting or staying active not only throughout extraordinary situations like the one caused by the COVID-19 outbreak, but also under common circumstances. Since physical activity can be seen as a cost-effective strategy for improving mental health, governmental and healthcare professionals’ actions are required to foster its implementation – not only to prevent the negative consequences of the current situation but also to protect people’s mental health in the long term.

## Data Availability

The dataset generated and analyzed during the current study is available from the corresponding author upon reasonable request.
